# RETRACTION: A Meta‐Analysis of the Effectiveness of Antibacterial Bone Cement in the Treatment of Diabetic Foot Skin Wound Infections

**DOI:** 10.1111/iwj.70185

**Published:** 2025-01-12

## Abstract

**RETRACTION**: WuS. , XuY. , GuoL. , and JiangX. , “A Meta‐Analysis of the Effectiveness of Antibacterial Bone Cement in the Treatment of Diabetic Foot Skin Wound Infections,” International Wound Journal 21, no. 3 (2024): e14487, 10.1111/iwj.14487.37973553 PMC10898415

The above article, published online on 16 November 2023, in Wiley Online Library (http://onlinelibrary.wiley.com/), has been retracted by agreement between the journal Editor in Chief, Professor Keith Harding; and John Wiley & Sons Ltd. Following an investigation by the publisher, all parties have concluded that this article was accepted solely on the basis of a compromised peer review process. In addition, the investigation found unattributed textual overlap between this article and multiple other articles by different authors (Huang, et al. 2023 [https://doi.org/10.1111/iwj.14286]); (Zhu, et al. 2023 [https://doi.org/10.1111/iwj.14279]); (Dong, et al. 2023 [https://doi.org/10.3389/fendo.2023.1134318]); and (Liu, et al. 2023 [https://doi.org/10.1111/iwj.14284]). The editors have therefore decided to retract the article. Authors L. Guo and Y. Xu have acknowledged that they were informed of the article's submission and acceptance but stated they were not involved in the data analysis, writing, submission, or revision of the submitted manuscript. Author Y. Xu has further stated that they have no objection to the retraction of the article. All other authors did not respond to the notice regarding the retraction.

**RETRACTION**: S. Wu , Y. Xu , L. Guo , and X. Jiang , “A Meta‐Analysis of the Effectiveness of Antibacterial Bone Cement in the Treatment of Diabetic Foot Skin Wound Infections,” International Wound Journal 21, no. 3 (2024): e14487, 10.1111/iwj.14487.37973553 PMC10898415

The above article, published online on 16 November 2023, in Wiley Online Library (http://onlinelibrary.wiley.com/), has been retracted by agreement between the journal Editor in Chief, Professor Keith Harding; and John Wiley & Sons Ltd. Following an investigation by the publisher, all parties have concluded that this article was accepted solely on the basis of a compromised peer review process. In addition, the investigation found unattributed textual overlap between this article and multiple other articles by different authors (Huang, et al. 2023 [https://doi.org/10.1111/iwj.14286]); (Zhu, et al. 2023 [https://doi.org/10.1111/iwj.14279]); (Dong, et al. 2023 [https://doi.org/10.3389/fendo.2023.1134318]); and (Liu, et al. 2023 [https://doi.org/10.1111/iwj.14284]). The editors have therefore decided to retract the article. Authors L. Guo and Y. Xu have acknowledged that they were informed of the article's submission and acceptance but stated they were not involved in the data analysis, writing, submission, or revision of the submitted manuscript. Author Y. Xu has further stated that they have no objection to the retraction of the article. All other authors did not respond to the notice regarding the retraction.

